# Dynamics of aggregate-associated organic carbon after long-term cropland conversion in a karst region, southwest China

**DOI:** 10.1038/s41598-022-27244-1

**Published:** 2023-01-31

**Authors:** Li Wen, Dejun Li, Kongcao Xiao, Haiming Tang, Chao Li, Xiaoping Xiao

**Affiliations:** 1 Hunan Soil and Fertilizer Institute, 730 Yuanda 2nd Road, Changsha, 410125 Hunan China; 2 Key Laboratory of Agro-Ecological Processes in Subtropical Region, Institute of Subtropical Agriculture, Chinese Academy of Sciences, Changsha, 410125 Hunan China; 3 Huanjiang Observation and Research Station for Karst Ecosystems, Institute of Subtropical Agriculture, Chinese Academy of Sciences, Huangjiang, 547100 Guangxi China

**Keywords:** Carbon cycle, Carbon cycle, Agroecology

## Abstract

Cropland conversion has a major impact on soil C sequestration. However, it remains unclear about the changes in soil aggregate and their contribution to C accumulation following cropland conversion in a karst region, southwest China. In this study, three different cropland use types (sugarcane, mulberry and forage grass cultivation) were selected to replace maize-soybean cultivation. The soil was collected at a depth of 0 to 30 cm for analysis of soil aggregates and their OC content. Results showed that macro-aggregate was the predominant component underlying four cropland use types. Forage grass cultivation remarkably increased the OC stock and aggregate stability (MWD and GMD). OC content and stock associated with aggregate varied with cropland use types and soil depth, but were typically highest in forage grass fields. Macro-aggregates contained higher OC content and stock than other aggregate fractions, along with soil depth underlying four cropland use types. The increases in OC stock in forage grass field was mainly due to increased OC stocks within macro-aggregates, which is further attributed to the increase in OC content within macro-aggregates. Overall, forage grass cultivation replaced maize-soybean cultivation was suggested as an ecological restoration model to enhance soil C sequestration potential, owing to its role in increasing OC stock of aggregation and aggregate stability, in the karst region of southwest China.

## Introduction

Soil is the largest carbon sink in terrestrial ecosystems, which is twice or three times as much as atmospheric and vegetation carbon (C) pool^1^. Accordingly, slight C losses from soil may lead to dramatic changes in atmospheric carbon dioxide level and subsequently accelerate global climate change^1,2^. Intensive agriculture based on cropland management practices is deteriorating soil quality, resulting in soil structure destruction, nutrient loss, and soil erosion^3^. Moreover, they can reduce soil C sequestration and accelerate greenhouse gas emissions^4,5^. Therefore, it is imperative to develop new restoration types to replace traditional intensive agriculture and reverse the process of soil C loss^6–8^.

Soil aggregation is a physical process that protects soil organic carbon (OC) by providing barriers between OC and OC decomposer, thus affecting the OC distribution and stability thereof^8–10^. Based on hierarchical model, soil aggregates consist of many fractions, mainly including macro-aggregates (> 0.25 mm) and micro-aggregates (< 0.25 mm)^11,12^. Generally, macro-aggregates formed from carbohydrate-rich plant residues and associated with soil organic matter (OM) increases^13,14^. Nevertheless, the soil OC within micro-aggregates decomposes slowly, which leads to a greater protection from microbial breakdown and ultimately to long-term C sequestration^15^. Thus, the formation of soil aggregates and soil C sequestration are mutually promoted processes^16,17^. Several studies have reported that soil C sequestration through soil aggregation responds to land use change more quickly than soil C within bulk soil^18–20^^.^ In agriculture erosion land, cropland conversion or restoration, often considered as an effective strategy to recuperate soil productivity and quality through enhancing soil aggregate stability and promoting soil C sequestration^21^. Furthermore, soil aggregate stability was an important indicator of soil physical quality, which could determine the magnitude and direction of soil C accumulation^3,19^. Therefore, understanding the pattern of soil aggregates following cropland conversion can provide insights into soil C sequestration and thus provide better land restoration strategies in degraded areas.

Soil OC stabilization in soil aggregate is the principal mechanism for soil C sequestration^14^. However, the contribution of associated-aggregate OC to bulk soil C accumulation or losses following cropland use change was not thoroughly understood. Soil aggregates are generally classified into macro-agggregate (> 2 mm), meso-aggregate (2–0.25 mm), micro-aggregate (< 0.25 mm) or silt–clay (< 0.053 mm) based on their size^22^, but they contributed to total soil C sequestration vary differently following cropland use change^19,23^. For example, Gelaw et al. (2015)^23^ reported that a 68.2% OC increase in afforested soil is occluded by soil macro-aggregates. While Zhong et al. (2019)^19^ demonstrated that soil OC stocks in meso-aggregte accounted for 46% of the total increase in bulk OC stocks when abandoned farmland 42 years ago converted to *Robinia pseudoacacia* with a combined 227.79% increase in total soil OC stocks at 0–20 cm depth. Furthermore, higher C mineralization rate has been observed in larger aggregate fractions than in smaller aggregate fractions^24,25^. Accordingly, the inconsistent conclusions of individual studies have added to the confusion surrounding the mechanisms that underlie the contribution of aggregates to total soil OC accumulation. Thus, it is essential to clarify which size of aggregates, and whether the aggregate amounts or the OC content associated with a specific aggregate fraction, are dominant modulators of OC accumulation in degraded agriculture ecosystems.

The karst area is widely distributed in the world and accounts for almost 12% of the earth’s land. In China, karst landscape covers about 0.55 million km^2^ in the southwest region^26^. Karst ecosystem was notorious for its role in land degradation, such as soil OM losses, soil degradation, and rocky desertification^27^. In the past decades, maize-soybean rotation (MS) was largely implemented in the karst region, which caused severe soil C loss and soil erosion^28^. Therefore, it is undoubtedly necessary to pay much attention to avoid further degradation of the existing cropland. Recently, several new conservation agriculture models, including sugarcane (SG), mulberry (MB) and forage grass (FG) cultivation, have been developed as new raising models to replace MS in the karst region^6,29^. Previous studies have reported that bulk soil OC change under cropland use change in karst region^6,7^, little is known about soil aggregate fractions and their contribution to bulk soil OC accumulation following cropland conversion. In the karst region, macro-aggregates generally prefer higher soil OC content than micro-aggregates following vegetation or cropland restoration^8,30^. Further research demonstrated that macro-aggregate contained much more OC stock than other size fractions^30,31^. Rationally, we hypothesized that the accumulation pattern of bulk soil OC was closely related to the changes of OC stock associated with macro-aggregates. The OC stocks within aggregates were predominantly determined by the amount of aggregates and its associated OC content^19,20^. Thus, the objective of this study was (1) to determine the distribution and stability of aggregates following cropland conversion, (2) to evaluate the OC content and stocks associated with aggregates following cropland conversion, and (3) to determine whether the changes in OC stocks aggregate-associated were primarily due to changes in the mass of aggregate fractions or to the changes in the OC content associated-aggregate.

## Materials and methods

### Site description

The study sites were located in Huanjiang County (24°44′–25°33′N, 107°51′–108°43′E) of Guangxi Zhuang Autonomous Region, southwest China. The detailed climate information was presented elsewhere^6,7,29^. The karst areas are characterized by gentle valleys embraced by hills and the soil is calcareous leptosols (limestone soil) based on the Food and Agriculture Union Organization World Reference Base for Soil Resources (International Union of Soil Sciences Working Group WRB, 2006)^7^.

The experiment adopted a randomized complete block design with four blocks (about 9 km^2^). With each block, one plot (greater than 400 m^2^ each) was selected for the fields of MS, MB, SG and FG. It is noted that the latter three types were converted from MS cultivation and had been under the same crop management for 15–20 years. Cropland uses were managed under regional typical practices. All of the information, i.e., crop species, tillage frequency, harvest frequency, fertilization rate and replanting frequency, have been described in previous studies^6,29^. In addition, the planted species in the forage grass field was hybrid Napier grass (*Pennisetum purpureum Schumach* × *P. amreicanum (L.) Leeke*). This grass is commonly considered as a perennial fodder crop because of its fast growth rate, drought tolerance, high productivity and protein content^32,33^. All plots were made available over the valley or bottom slopes. In total, 16 plots (four plots × four cropland use types) were included.

### Soil sampling and analysis

Soil samplings were conducted in October 2019. In each plot, we collected four soil profiles (0–30 cm), randomly using soil cores with possible litters removed. Each profile was divided into three horizons, that is, 0–10 cm, 10–20 cm and 20–30 cm. In addition, two pits of 60 × 60 × 30 cm were dug to collect samples using metal rings (volume:100 cm^3^, SYK-01, China) for the measurement of bulk density at the three horizons. After picked out the possible stones and roots. Soil samples were air-dried, gently crushed and divided into two portions for analysis, including (1) one was passed through 2 mm sieve for determining OC content of bulk soil and (2) aggregation fractions and their OC contents.

### Aggregate fractionation

The soil aggregate fractions were determined by dry sieving methods, as described by Gartzia-Bengoetxea et al. (2009)^34^. Briefly, 100 g of air-dried soil samples were taken on a nest of sieves (2 mm, 1 mm, 0.5 mm and 0.25 mm), then agitated for 90 s with a sieve shaker at 600 oscillation min^−1^. The soil retained on each sieve and in the bottom container was collected under 0.25 mm sieve. The soil aggregates were separated as macro-aggregates (> 2 mm), meso-aggregates (2–0.25 mm), and micro-aggregates (< 0.25 mm). To determine the mass distribution of soil fractions, the sub-sample of soil fractions were oven dried at 105 ℃ and weighted at room temperature. A special aggregate sample and bulk soil was ground to pass through a 100 mesh sieve to measure soil OC content by wet oxidation with dichromate redox colorimetric method^35^. Mean weight diameter (MWD) and geometric mean diameter (GMD) of soil aggregates were calculated as by the following Eq. ^23^:

1 $${\text{MWD}} = \sum\limits_{i = 1}^{n} {\overline{{X_{i} }} } W_{i}$$

2 $${\text{GMD}} = {\text{Exp}}\left( {\sum\limits_{i = 1}^{n} {W_{i} \ln \overline{{X_{i} }} } } \right)$$

where n is the number of aggregates ranges, X_i_ is the mean diameter of each size fraction, and W_i_ is the proportion of each size to the total sample (%).

The stocks of OC (Mg C hm^−2^) in bulk soil were calculated using the following equation:

3 $${\text{OC stock in bulk soil}} = \frac{{{\text{OC content }} \times {\text{D}} \times {\text{BD}}}}{10}$$

where D is the thickness (cm) of the soil layer, BD is the bulk density (g cm^−3^) and OC is the content (g kg^−1^).

The stocks of organic carbon in aggregates fractions were calculated using the following equation:

4 $${\text{M}}_{{\text{i}}} = \frac{{{\text{D}} \times {\text{BD}} \times {\text{W}}_{{\text{i}}} }}{10}$$

5 $${\text{Stock of OC}}_{{\text{i}}} = {\text{M}}_{{\text{i}}} \times {\text{OC}}_{{\text{i}}}$$

where Mi is the amount of soil in the i_th_ size fraction (kg m^−2^) and OC_i_ is the OC content of the i_th_ size fraction (g kg^−1^). and W_i_ is the proportion of the total soil in the i_th_ size fraction (%). Additionally, we applied the procedure recommended by Zhong et al. (2019)^19^ and Wei et al. (2013)^20^ to evaluate the relative contribution of changes in aggregate mass and aggregated-associated OC contents to the total changes in OC stocks within each aggregate fraction. We assumed that changes in OC stock within any particular aggregate fraction were induced both changes in OC content of aggregate fraction (F1) and by changes in the mass of aggregate fraction (F2). Hence, the F1 and F2 were calculated using the following equations:

6 $${\text{F}}1 = {\text{M}} \times \Delta {\text{C}}$$

7 $${\text{F}}2 = \Delta {\text{M}} \times {\text{C }}$$

where F1 is the change in OC stock (Mg C hm^−2^) aggregate associated due to changes in OC content associated with aggregate, F2 is the change in OC stock within an aggregate fraction due to changes in the mass of aggregate fraction, and M is the initial mass of the aggregate fraction in MS, ΔM is the change in the mass of a particular fraction after cropland conversion, C is the final OC content of the aggregate fraction (g kg^−1^) after conversion, and ΔC is the change in the OC content of the aggregate fraction (g kg^−1^) caused by cropland conversion.

### Statistical data analysis

A two-way analysis of variance (ANOVA) was conducted using SPSS 21.0 (SPSS, Inc., Chicago, IL, USA) to test the effects of cropland use types and soil depth on (i) OC content and stock in bulk soil, (ii) aggregate fraction distribution, and (iii) OC content and stocks associated with aggregate. *Person’s* correlation analysis was performed to identify the relationships between OC stocks in bulk soil and the proportion of aggregate and its OC content and stock. The difference was considered significant if the *p* value was less than 0.05.​

### Ethics approval and consent to participate

All the crop plants in this study were obtained from the local economical crop. Corn, soybean, sugarcane, mulberry and forage grass were not wild and endangered plants, and all the crop experiments were in compliance with relevant institutional, national and international guidelines and legislation.

## Results

### OC content and stocks in bulk soil

**Figure 1:**
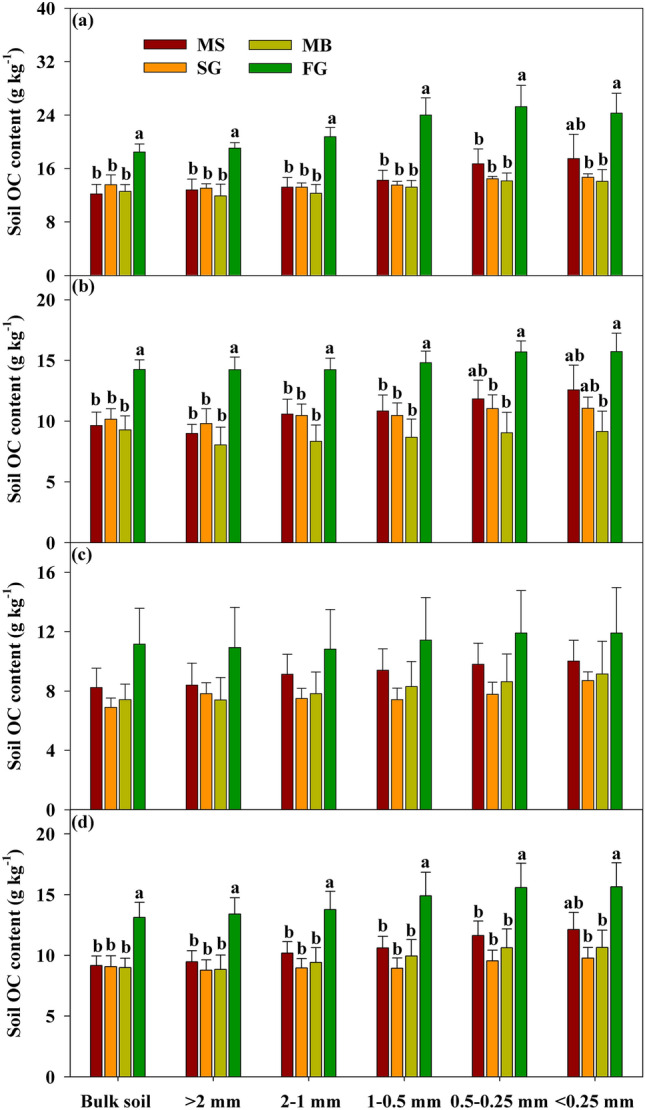
OC contents of bulk soil and aggregate fractions under four land use types at (**a**) 0–10 cm, (**b**)10–20 cm, (**c**) 20–30 cm, and (**d**) 0–30 cm, respectively. Values presented as means with standard error. Different letters indicate significant difference at *p* < 0.05 among four cropland use types. MS: maize-soybean; SG: sugarcane; MB: mulberry; FG: forage grass.

**Figure 2:**
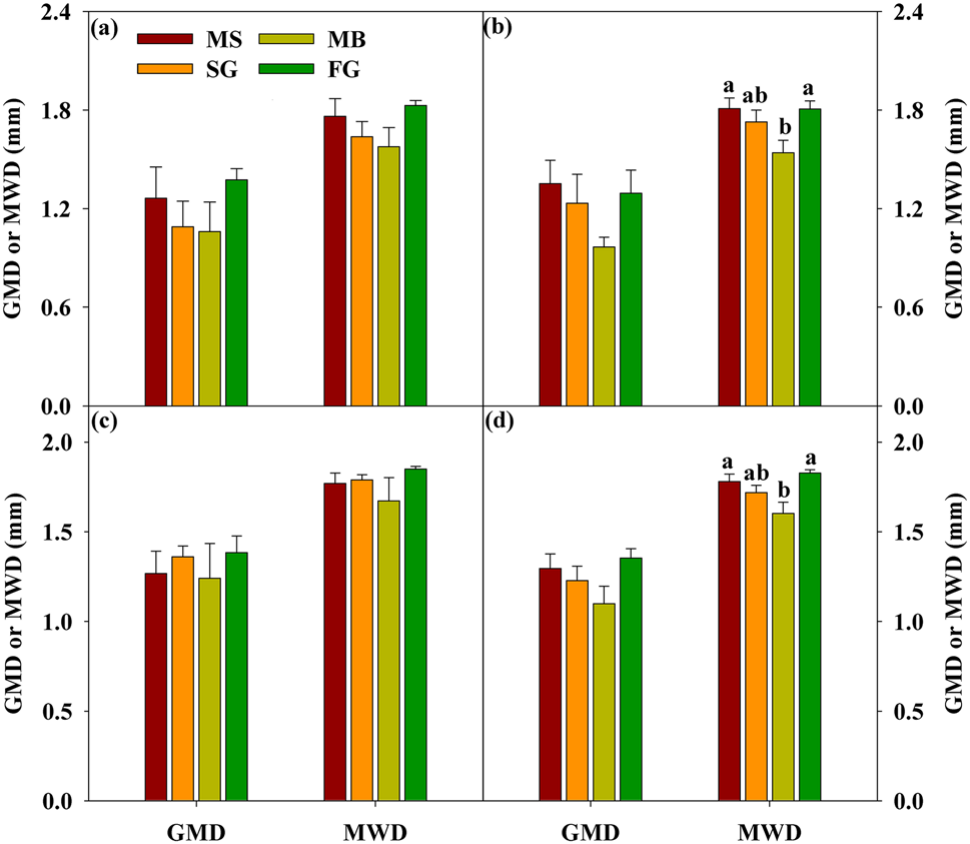
The value of GMD and MWD of soil aggregates at (**a**) 0–10 cm, (**b**) 10–20 cm, (**c**) 20–30 cm, and (**d**) 0–30 cm, respectively, under four cropland use types. Values presented as means with standard error. Different letters indicate significant difference at *p* < 0.05 among four cropland use types. MS: maize-soybean; SG: sugarcane; MB:mulberry; FG: forage grass.

According to two-way ANOVA, cropland use type and soil depth had significant influence on OC content in bulk soil, respectively (Table 1, *p* < 0.01). The highest value of OC content and stock in bulk soil was found in FG regardless soil depth, while that in SG and MB was not significantly differed from that in MS (Fig. 1, 2). Specially, compared to MS, OC content in FG increased by 51.87%, 47.98% and 35.48%, OC stock increased by 44.80%, 53.91% and 30.54%, at 0–10 cm, 10–20 cm and 20–30 cm, respectively (Figs. 1, 2). Further analysis indicated that OC content and stock in bulk soil was significantly decreased with soil depth under four cropland use types (Table 1, S1).

**Table 1:** The two-way ANOVA results for all variables.

Soil variables	Cropland use type		Soil depth		Cropland use type $\times$ Soil depth	
	F	*P*	F	*P*	F	*P*
MWD	4.25	** < 0.01**	0.77	0.47	0.33	0.92
GMD	1.66	0.19	0.74	0.492	0.41	0.87
Aggregate size distribution						
> 2 mm	2.34	0.09	0.92	0.41	0.41	0.86
2–1 mm	0.21	0.89	0.74	0.48	0.46	0.83
1–0.5 mm	0.98	0.41	0.80	0.46	0.41	0.87
0.5–0.25 mm	2.91	**0.05**	0.53	0.59	0.26	0.95
< 0.25 mm	6.02	** < 0.01**	0.47	0.63	0.17	0.98
OC content						
Bulk soil	14.26	** < 0.01**	21.67	** < 0.01**	0.43	0.85
> 2 mm	14.38	** < 0.01**	17.24	** < 0.01**	0.13	0.99
2–1 mm	16.15	** < 0.01**	21.84	** < 0.01**	0.76	0.61
1–0.5 mm	16.01	** < 0.01**	22.51	** < 0.01**	1.20	0.33
0.5–0.25 mm	12.36	** < 0.01**	21.77	** < 0.01**	0.79	0.59
< 0.25 mm	7.58	** < 0.01**	13.69	** < 0.01**	0.42	0.86
OC stock						
Bulk soil	16.23	** < 0.01**	22.32	** < 0.01**	0.85	0.54
> 2 mm	13.74	** < 0.01**	5.95	** < 0.01**	0.58	0.74
2–1 mm	2.52	** < 0.01**	4.76	**0.02**	0.23	0.96
1–0.5 mm	0.05	0.98	4.30	**0.02**	0.25	0.95
0.5–0.25 mm	0.92	0.44	4.04	**0.03**	0.30	0.93
< 0.25 mm	2.91	** < 0.05**	4.33	**0.02**	0.27	0.95

Bold values were significant at *p* < 0.05. MWD: mean weight diameter. GWD: geometric mean diameter.

Significant values are in [bold].

### Aggregate distribution and its stability

Among the five aggregate fractions, macro-aggregates (> 2 mm) dominated the majority portion (77.14 ± 1.4%, averagely) of total soil, whereas micro-aggregates (< 0.25 mm) accounted the lowest proportion (4.6 ± 0.52%, averagely) (Table 2). Cropland conversion significantly impacted the amount of meso-aggregates (0.5–0.25 mm) and micro-aggregates (< 0.25 mm), which was significantly higher in MB, followed by SG, and lower in MS and FG (Table 1, 2, *p* < 0.05). Furthermore, the proportion of macro-aggregates was significantly increased while that of meso-aggregates (0.5–0.25 mm) and micro-aggregates decreased significantly when MS converted to FG overall (Table 2, *p* < 0.05), and no significant differences in the proportion of above mentioned fractions were detected in SG and MB fields (Table 2, *p* > 0.05).

**Table 2:** Distribution of aggregate fraction (%) under four cropland use types.

	Size aggregate				
	> 2 mm	2–1 mm	1–0.5 mm	0.5–0.25 mm	< 0.25 mm
0–10 cm					
MS	76.50 (8.60)	12.13 (2.99)	3.63 (1.65)	2.93 (1.66)	4.80 (2.77)
SG	68.73 (7.80)	12.20 (2.66)	5.03 (1.81)	4.85 (1.82)	9.18 (1.67)
MB	66.95 (7.73)	10.25 (2.54)	3.93 (1.17)	5.55 (2.21)	13.30 (4.75)
FG	82.63 (2.62)	9.50 (1.59)	2.18 (0.59)	1.90 (0.63)	3.80 (0.77)
10–20 cm					
MS	81.08 (5.19)	9.93 (1.85)	2.63 (0.96)	2.18 (0.93)	4.15 (1.50)b
SG	75.43 (6.70)	10.68 (2.99)	3.70 (1.33)	3.40 (1.24)	6.80 (1.66)b
MB	64.10 (3.73)	11.10 (1.48)	4.33 (0.90)	6.10 (2.37)	14.40 (4.16)a
FG	79.53 (5.28)	11.97 (3.16)	2.70 (1.08)	1.90 (0.72)	3.87 (1.25)b
20–30 cm					
MS	77.73 (4.73)	11.20 (1.57)	3.33 (0.86)	2.95 (0.96)	4.73 (1.52)
SG	81.55 (3.10)	7.83 (0.90)	2.48 (0.30)	2.45 (0.23)	5.70 (1.20)
MB	74.83 (8.15)	7.65 (0.78)	2.95 (0.90)	4.13 (2.04)	10.45 (5.55)
FG	83.27 (2.59)	10.40 (3.39)	2.07 (0.27)	1.50 (0.30)	2.77 (1.08)
0–30 cm					
MS	78.43 (3.40)	11.08 (1.19)	3.19 (0.64)	2.68 (0.65)b	4.56 1.06)b
SG	75.23 (3.54)	10.23(1.35)	3.73 (0.75)	3.57 (0.73)ab	7.23 (0.91)b
MB	69.04 (4.07)	9.54 (1.05)	3.68 (0.56)	5.18 (1.17)a	12.56 (2.65)a
FG	81.89 (1.87)	10.51 (1.37)	2.30 (0.37)	1.78 (0.31)b	3.51 (0.54)b

Data are presented by means with standard error. Different letters indicate significant difference among four cropland use types at *p* < 0.05. MS: maize-soybean; SG: sugarcane; MB:mulberry; FG: forage grass.

**Figure 3:**
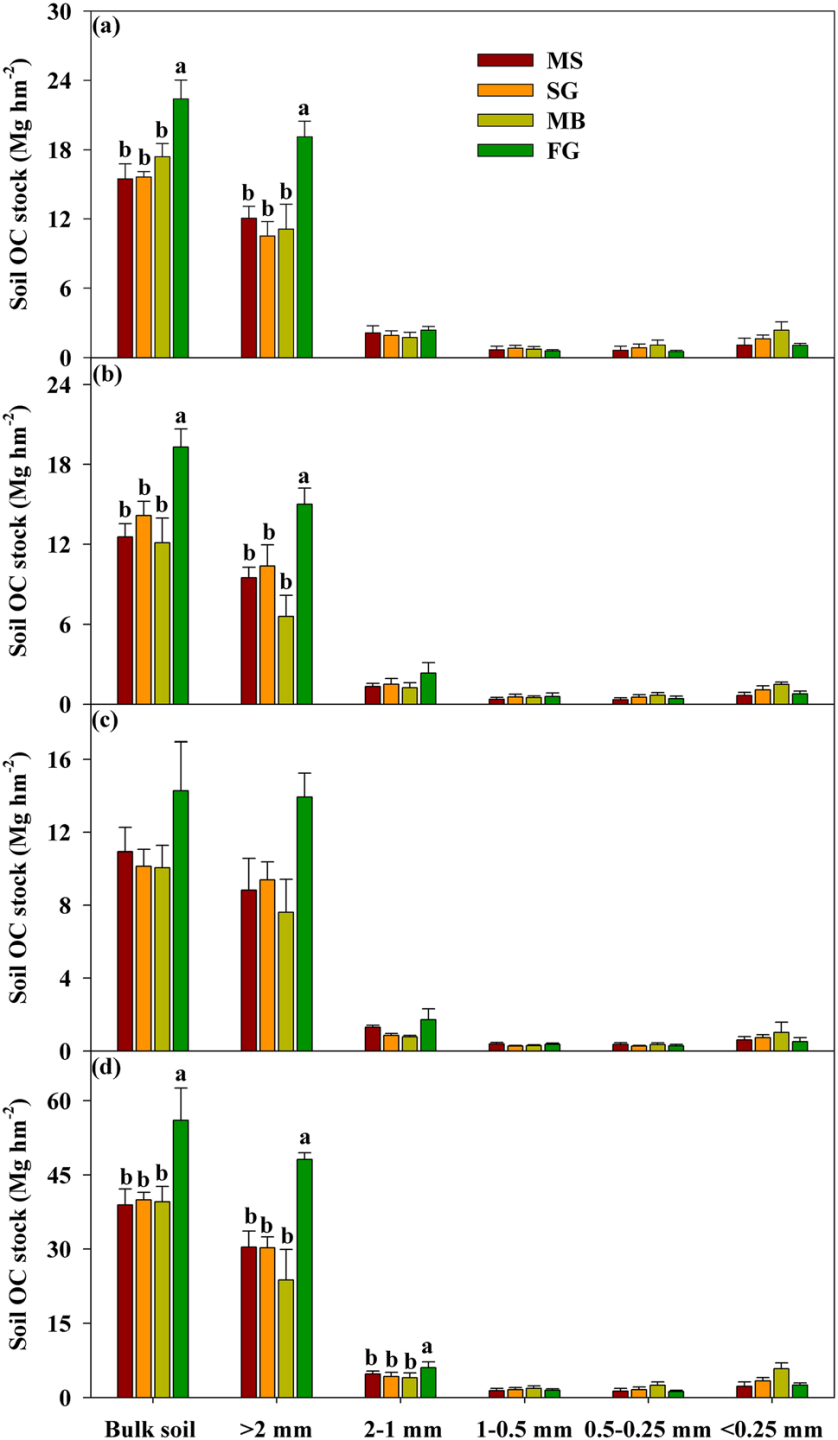
OC stocks of bulk soil and aggregate fractions under four land use types at (**a**) 0–10 cm, (**b**) 10–20 cm, (**c**) 20–30 cm, and (**d**) 0–30 cm, respectively. Values presented as means with standard error. Different letters indicate significant difference at *p* < 0.05 among four cropland use types. MS: maize-soybean; SG: sugarcane; MB: mulberry; FG: forage grass.

The value of MWD was significantly affected by cropland use type (Table 1, *p* < 0.01). The MWD value was significantly decreased following the conversion from MS to MB, and there was no significant difference among MS, SG and FG, especially at 10–20 cm soil layer (Fig. 3). The GMD value was not impacted by cropland use type and soil depth, which varied from 0.72 to 1.73 under four cropland types across 0–30 cm soil layers (Fig. 3).

### OC content and stocks associated with aggregate fractions

Both cropland use type and soil depth had significant influence on the OC content associated with aggregates (Table1, *p* < 0.05). However, these indexes were not affected by the interactions between cropland use type and soil depth (Table 1, *p* > 0.05). OC content within five size fractions were significantly declined with soil depth (Table 1, S1, *p* < 0.05). Cropland conversion had a significant effect on OC content associated-aggregate at 0–10 cm and 10–20 cm, which were significantly higher in FG fields compared to the other three cropland use types (Fig. 1a–b, *p* < 0.05). For detail, After cropland conversion from MS to FG, OC content of > 2 mm, 2–1 mm, 1–0.5 mm, 0.5–0.25 mm and < 0.25 mm size fractions increased by 47.08%, 40.18%, 47.33%, 39.33% and 30.66%, respectively (Fig. 1d). Nevertheless, soil OC content associated-aggregate remained consistent at 20–30 cm soil layer across four cropland use types (Fig. 1c, *p*  > 0.05).

The OC stock with macro-, meso- and micro-aggregates fractions, like OC content, showed a clear decreasing trends with soil depth (Table S1, *p* < 0.05). Whereas, cropland use change only significantly affected the OC stock with macro-, 2–1 mm and micro-aggregate fractions (Table 1, *p* < 0.05). For instance, OC stocks associated with macro- and 2–1 mm aggregate fractions were significantly increased when MS converted into FG (Fig. 2d, Table S1, *p* < 0.05). At 0–10 cm and 10–20 cm, FG cultivation significantly increased the OC stock with macro-aggregates. And at 20–30 cm layer, the higher value of OC stock was found in macro-,2–1 mm aggregate fractions in FG (Fig. 2d, *p* < 0.05). Furthermore, there was no significant variation in OC stocks associated with the above mentioned aggregate fractions detected in MS, SG and MB soils regardless of soil depth (Fig. 2, *p* > 0.05).

The increase in OC stocks aggregate-associated was varied different following cropland conversion (Table S2). Compared with MS field, the highest increment of OC stocks within macro-aggregate was observed in FG field (increased by 7.05, 5.51 and 5.10 Mg hm^−2^ at 0–10 cm, 10–20 cm, and 20–30 cm, respectively) (Table S2). Further analysis indicated that the highest incrementary ratio in OC stock within macro-aggregate was observed in FG field at 0–10 cm soil layer (Table S2).

### Contributions of the changes in aggregates dynamics to soil OC sequestration

**Figure 4:**
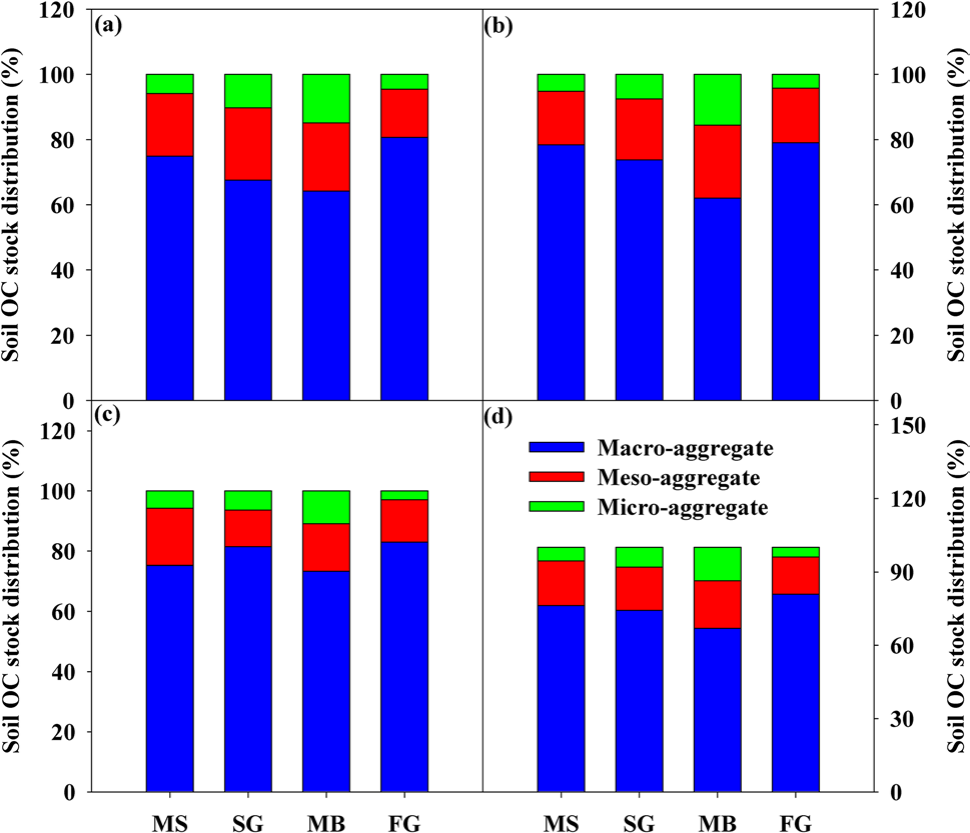
The relative distribution of OC stocks in different soil aggregate fractions at (**a**) 0–10 cm, (**b**) 10–20 cm, (**c**) 20–30 cm and (**d**) 0–30 cm), respectively. Each point represents mean value. MS: maize-soybean; SG: sugarcane; MB: mulberry; FG: forage grass. Macro-aggregate: > 2 mm; Meso-aggregate: 2–0.5 mm; Micro-aggregate: < 0.25 mm.

The percentage of OC stock associated-aggregate varied in different aggregate fractions following cropland conversion (Fig. 4). The majority of OC stock was contained in macro-aggregate (67.2–92.3%), followed by meso-aggregate and micro-aggregate underlying four cropland use types across 0–30 cm soil layer (Fig. 4). Moreover, the proportion of OC stock associated with macro-aggregate was higher in FG than in other three cropland use types (Fig. 4).

**Figure 5:**
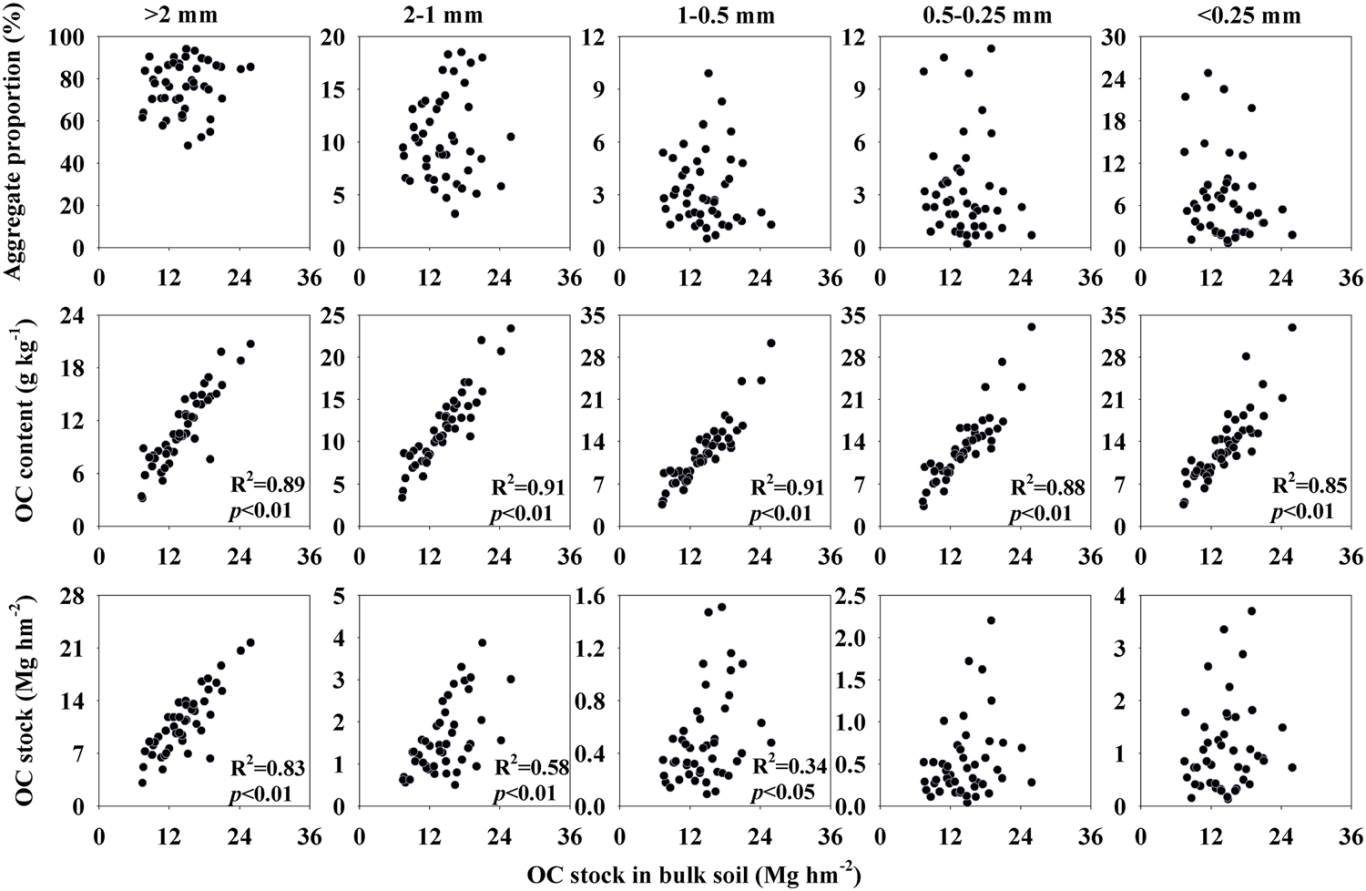
Correlations between the proportion, OC content and stock associated with aggregates and OC stocks in bulk soils (0–30 cm).

The dynamics of contribution of the changes in associated aggregates to OC stock were presented in Table 3. Our results showed that the OC content associated with aggregates and OC stocks associated with macro-aggregate and 2–0.5 mm size class were positively correlated with OC stocks of bulk soil (Fig. 5, *p* < 0.05). In accordance with the results of linear regression analysis, the accumulation pattern of OC stocks in bulk soil following cropland conversion is mainly due to the changes of OC content associated with macro-aggregates, further indicated that the values of F1 and F2 were greatly higher in macro-aggregates than other four size classes, regardless of depth (Table 3). Moreover, the absolute value of F1 and F2 in FG field were distinctly higher than those in SG or MB field with minimal exception (Table 3).

**Table 3:** Changes in the OC stocks associated with five aggregate fraction after the replacement of MS to SG, MB and FG.

		SG		MB		FG	
	Soil depth (cm)	F1	F2	F1	F2	F1	F2
> 2 mm	0–10	0.24	− 11.1	− 0.66	− 10.15	6.33	− 15.05
	10–20	0.9	− 9.37	− 0.85	− 7.82	5.52	− 13.02
	20–30	− 0.72	− 7.52	− 0.89	− 7.09	5.15	− 12.15
2–1 mm	0–10	− 0.06	− 0.29	− 0.17	− 0.34	0.83	1.15
	10–20	− 0.09	− 0.28	− 0.33	− 0.39	0.64	0.14
	20–30	− 0.19	− 0.58	− 0.2	− 0.53	0.55	− 0.24
1–0.5 mm	0–10	− 0.08	1.19	− 0.07	1.15	0.21	4.82
	10–20	− 0.03	0.75	− 0.16	0.48	0.17	1.67
	20–30	− 0.07	0.22	− 0.11	0.39	0.12	1.38
0.5–0.25 mm	0–10	− 0.15	1.53	− 0.27	1.49	0.13	5.71
	10–20	− 0.05	0.92	− 0.38	0.6	0.11	2.01
	20–30	− 0.07	0.3	− 0.25	0.49	0.09	1.56
< 0.25 mm	0–10	− 0.33	1.21	− 1.04	1.16	0.24	4.6
	10–20	− 0.14	0.61	− 0.75	0.37	0.13	1.62
	20–30	− 0.1	0.19	− 0.51	0.37	0.16	1.24

F1 present the change in OC stock attributed to changes in the OC content associated-aggregate following cropland conversion. F2 present the change in OC stock attributed to changes in the mass of aggregate fraction after conversion. SG: sugarcane; MB: mulberry; FG: forage grass.

## Discussion

### Effects of cropland conversion on OC pool in bulk soil

Cropland restoration identified as an efficient ecological project to promote soil C sequestration in karst erosion areas^28,30^. The conversion from MS to FG resulted in the total soil OC content and stock across 0–30 cm layers increasing by 46.12% and 43.73% respectively. The result was highly coincident with previous studies observed at 0–10 cm layer, which reported that FG cultivation replaced from MS cultivation could remarkably increase soil OC pool in karst region, Southwest China^28^. In our study, the lower OC content and stock in MS may be partially attributed to the non-returned crop residues and increased exposure of deep soil OM to oxygen under tillage disturbance, resulting in decreased soil OC accumulation through reducing the input of OM and accelerating OM decomposition^28,30,37,38^. Nevertheless, the conversion from MS to FG can increase the soil OC pool by increasing inputs from crops. For detail, laregly aboverground crops are harvested and removed from the fields each every year for economic production, there is thus a lack of aboverground OC input. Therefore, the root biomass became the main source of OM inputs, and even slight changes in biomass can substantially alter soil C level^39^. In the present study, the root biomass in FG field was approximately 6 times that in MS field (110.06 ± 17.24 kg hm^−2^ averagely) (Table S2). Consequently, the higher root biomass in FG are responsible for the corresponding higher C storage of fine root in FG, which is supported by the fact that higher amount of C were stored in the fine roots of FG field compared with that of MS field (Table S2). In fact, several studies have demonstrated that cultivation of perennial grasses is efficient in stimulating soil OC accumulation owing to its great amount of fine roots and underground biomass^33,40^. Soil disturbance (such as tillage) is one of the main causes of soil C depletion in agricultural systems, and increased tillage practice can result in greater soil C loss^41–43^. Therefore, the frequent tillage conducted in MS field resulted in lower levels of OC than that in FG field under minimal tillage disturbance.

### Impacts of cropland conversion on soil aggregates structure and stability

Soil structure plays an important role in soil environment and quality, which is strongly characterized by soil aggregates and their stability^43,44^. In our study, soil macro-aggregates dominated the largest portion of total soil while meso-aggregates and micro-aggregates were only accounted for a small portion, indicating that cropland conversion could facilitated the formation of macro-aggregates (Table 2). These findings are in line with other studies, wherein that macro-aggregates occupied the major portion of total soil following farmland or vegetation restoration^19,30^. Tillage disturbance often disrupts aggregates by bringing subsurface soil to the surface, which can readily promote soil C turnover and hinder macro-aggregate formation^45^. Conversely, minimal tillage experienced and greater accumulation of root residues resulted in higher C accumulation in the FG field. Furthermore, fine roots improved the soil aggregate stability via the interaction with mycorrhizal fungi, which produced exudates and binding agents and promoted the formation of soil aggregates^46,47^. Therefore, higher inputs of root residue in the soil could enhance the capacity of aggregate re-formation. In fact, these can be supported by the higher value of root biomass and its C stock in the FG field. In addition, forage grass cultivation can enhance the formation of large and stable soil aggregates by fine roots and fungal hyphae through the production of exudates and binding agents, such as humic compounds, polymers and roots^48,49^. Thus, few tillage disturbance and higher inputs of root biomass in FG field resulted in soil aggregation enhanced, especially macro-aggregates.

Soil aggregate stability can also be characterized by the values of MWD and GMD. Higher MWD or GMD values indicate greater aggregate stability due to more agglomerate ability. The value of MWD in the current study varied from 1.36 to 1.96, which was classified as “stable” by LeBissonnais' categorization of aggregate stability^50^.

Regardless of soil depth, the FG field had the greatest MWD and GMD values, indicating that its soil aggregates were more stable than those of the other three cropland use types. We may thus draw the conclusion that FG cropland conversion can improve the stability of aggregates based on MWD and GMD.

### Changes in OC stocks associated –aggregates following cropland conversion

Cropland use change generally affects soil C sequestration through changing OM inputs and decomposition^19^. Our study revealed that aggregate-associated OC was significantly higher in FG field than in MS field. These increases were mainly attributed to the new C derived from root residues inputs and decreased losses of OC associated-aggregate by C mineralization in FG soil^49^. Generally, tillage can breakdown large aggregates into small aggregates, and thus decrease the formation of soil macro-aggregates^41,42^. Thus, the lower OC content and stock associated-aggregate in MS field can be attributed to the OC loss resulting from soil erosion, and OM input reduction with tillage disturbance^8,30,45^.

In this study, the effects of cropland conversion on OC content associated-aggregate fractions occurred in the top 20 cm soil layers. In the karst region, approximate 57–89% of crop roots are concentrated in the surface soil layer, which directly affects OM inputs from underground root residues^51,52^. Meanwhile, tillage practices also happened on top 20 cm soil layer^6,28,29^. As a result, in soils below 20 cm, little or no tillage disturbance and limited OM inputs resulted in fewer or no distinctly changing levels of OC content associated with aggregate following cropland use change.

Cropland use change not only affected the OC stocks in bulk soil, but also affected the OC stocks associated-aggregates (Table 1). The difference of sensitivity of OC associated-aggregate to cropland use change may affect its contribution to bulk soil OC accumulation^30,38^. In our study, the macro-aggregate fraction was the most important contributor to total OC stock increase, followed by meso-aggregate and micro-aggregate (Fig. 4). This is primarily due to the higher amount and OC content of macro-aggregates. Overall all cropland use types, the OC stock associated with macro-aggregate in FG field was higher than that in other three cropland types regardless of soil depth (Fig. 4). For instance, OC stocks within macro-aggregate accounted for about 85.40%, 77.72% and 97.55% of total soil OC stock at 0–10 cm, 10–20 cm and 20–30 cm, respectively, under the conversion from MS to FG. Thus, the accumulation pattern of bulk soil OC stocks could closely related with changes of OC stocks associated with macro-aggregate under cropland use change.

The physical protection of OC in aggregates is regarded as one of the main mechanisms for soil OC accumulation through diminishing soil OC degradation and preventing its interaction with mineral particles^53,54^. In the present study, OC stock in bulk soil correlated substantially with the OC content-associated aggregate following cropland conversion (Fig. 5). Further analysised revealed that OC stocks in bulk soil was significantly correlated to OC stock associated with macro-aggregate (R^2^ = 0.83, *p* < 0.01), confirming that macro-aggregates are the major contributor to bulk soil OC accumulation where most OC stocks contained (Fig. 5). These may be caused by the binding agents of macro-aggregate, such as fungal hyphae, mycorrhizal hyphae, bacterial cells, and algae, which are highly dependent on soil OC and develop simultaneously with crop growth and build up a visible organic skeleton to enmesh the mineral particles by adsorption to form young macro-aggregates^15,55,56^. These patterns can proven by the shift in the increment of soil OC stocks following cropland conversion. Our calculation showed that the incrementary ratio of OC stock associated with macro-aggregate was more than 58% when MS replaced by FG (Table S3). However, the increment of OC stock associated with macro-aggregate in SG and MB field was relatively smaller and even shows a declining trend (Table S3). Based on the theory of hierarchical aggregation, OC stock increases with increasing aggregate size because of larger aggregates are composed of small particles plus organic binding agents^15,29^. Therefore, OC stocks in macro-aggregate were higher than those in other size fractions (Fig. 4). Overall, our findings demonstrate that OC accumulation was mainly due to the contribution of macro-aggregates. In the title of Table S3, the unit "Mg/hm2" changed "Mg/hm^2^".

Changes of OC stocks within aggregates are mainly attributed to two factors: (1) changes in the mass of special aggregate fractions and (2) changes in the OC content associated-aggregate fractions^19,20^. Thus, a better understanding of the dynamic of soil OC pool response to cropland use change is needed to character the changes of mass and OC content within special soil aggregates. Based on our calculation, the contribution pattern of aggregate mass and its OC content to the net accumulation of OC stock within aggregates differed substantially among four cropland use types. Specifically, the increase in OC stock within macro-aggregate was primarily attributed to increases in the OC contents of macro-aggregate and decreases in the mass of macro-aggregate fraction when MS converted to FG (Table 3). Prior work in karst area have reported that a relative higher recovery of soil aggregate structure and larger increase in macro-aggregate amount following vegetation restoration, indicating that OC accumulation relies on a well-developed soil structure^30^. These changes were also supported by the fact that the value of F1was greater than F2 in macro-aggregate when MS converted to FG (Table 3). Nevertheless, slight decreases in both mass and its OC content associated-aggregate fractions result in few or no changes in OC stock of macro-aggregate when MS converted to SG or MB (Table 3). Therefore, we can conclude that MS converted to FG can effectively improve the bulk soil OC stock in the karst region of southwest China. The OC stocks associated with macro-aggregate contribute to the most to increase bulk soil OC accumulation, which depends on the OC content within macro-aggregate following the conversion from MS to FG.

## Conclusions

The results of this study indicate that cropland conversion has a significant impact on the aggregate size proportion and OC stock in bulk soil and aggregate fractions. Macro-aggregates (> 2 mm) were predominant in all cropland use types. The conversion from MS into FG, substantially increase the proportion of soil macro-aggregate and its OC content, resulting in OC accumulation in the bulk soil and macro-aggregates. Aggregate stability (MWD and GMD was higher in FG than other three cropland use types, indicating a higher stability and aggregation under FG field. The increase in OC stocks within macro-aggregate was the major contributor for the increase in bulk soil OC stock, which depends on the increase of OC content within macro-aggregate. Overall, in karst region, FG replaced from MS was suggested as an ecological restoration model to enhance soil C sequestration potential, owing to its role in increasing OC stock of aggregation and aggregate stability.

## Supplementary Information

Supplementary Tables.

## Data Availability

The datasets used and/or analysed during the current study are available from the corresponding author on reasonable request.

## References

[1] Lal R (2004). Soil carbon sequestration impacts on global climate change and food security. Science (New York N.Y.).

[2] Deng L (2016). Effects of age and land-use changes on soil carbon and nitrogen sequestrations following cropland abandonment on the Loess Plateau China. Ecol. Eng..

[3] Jat HS (2019). Effects of tillage, crop establishment and diversification on soil organic carbon, aggregation, aggregate associated carbon and productivity in cereal systems of semi-arid Northwest India. Soil Tillage Res..

[4] Xie H (2018). Does intensive land use promote a reduction in carbon emissions? Evidence from the Chinese industrial sector. Resour. Conserv. Recycl..

[5] Liang LL, Grantz DA, Darrel Jenerette G (2016). Multivariate regulation of soil CO_2_ and N_2_O pulse emissions from agricultural soils. Global Change Biol..

[6] Li D (2018). Forage grass cultivation increases soil organic carbon and nitrogen pools in a karst region, southwest China. Land Degrad. Dev...

[7] Wen L (2017). Dynamics of soil organic carbon in density fractions during post-agricultural succession over two lithology types, southwest China. J. Environ. Manag..

[8] Xiao L (2021). The formation of large macroaggregates induces soil organic carbon sequestration in short-term cropland restoration in a typical karst area. Sci. Total Environ..

[9] Lützow MV (2006). Stabilization of organic matter in temperate soils: mechanisms and their relevance under different soil conditions–a review. Eur. J. Soil Sci..

[10] Amézketa E (1999). Soil aggregate stability: a review. J. Sustain. Agric..

[11] Six J (1998). Aggregation and soil organic matter accumulation in cultivated and native grassland soils. Soil Sci. Soc. Am. J..

[12] Cambardella CA, Elliott ET (1993). Carbon and nitrogen distribution in aggregates from cultivated and native grassland soils. Soil Sci. Soc. Am. J..

[13] Tisdall JM, Oades JM (1980). The effect of crop rotation on aggregation in a red-brown earth. Soil Res..

[14] Verchot LV (2011). Organic matter stabilization in soil aggregates: understanding the biogeochemical mechanisms that determine the fate of carbon inputs in soils. Geoderma.

[15] Six J, Elliott E, Paustian K (2000). Soil macroaggregate turnover and microaggregate formation: a mechanism for C sequestration under no-tillage agriculture. Soil Biol. Biochem..

[16] Bronick CJ, Lal R (2005). Soil structure and management: a review. Geoderma.

[17] Liu H (2020). and Zhang, Effects of agricultural abandonment on soil aggregation, soil organic carbon storage and stabilization: results from observation in a small karst catchment southwest China. Agric. Ecosyst. Environ..

[18] Zhu G, Shangguan Z, Deng L (2017). Soil aggregate stability and aggregate-associated carbon and nitrogen in natural restoration grassland and Chinese red pine plantation on the Loess Plateau. Catena.

[19] Zhong Z (2019). Effects of land use change on organic carbon dynamics associated with soil aggregate fractions on the Loess Plateau China. Land Degrad. Dev..

[20] Wei X (2013). Dynamics of aggregate-associated organic carbon following conversion of forest to cropland. Soil Biol. Biochem..

[21] Tang FK (2016). Effects of vegetation restoration on the aggregate stability and distribution of aggregate-associated organic carbon in a typical karst gorge region. Solid Earth.

[22] von Lützow M (2007). SOM fractionation methods: relevance to functional pools and to stabilization mechanisms. Soil Biol. Biochem..

[23] Gelaw AM, Singh BR, Lal R (2015). Organic carbon and nitrogen associated with soil aggregates and particle sizes under different land uses in tigray Northern Ethiopia. Land Degrad. Dev..

[24] Rabbi SM (2014). Soil organic carbon mineralization rates in aggregates under contrasting land uses. Geoderma.

[25] Mustafa A (2020). Soil aggregation and soil aggregate stability regulate organic carbon and nitrogen storage in a red soil of southern China. J. Environ. Manag..

[26] Jiang Z, Lian Y, Qin X (2014). Rocky desertification in southwest China: impacts, causes, and restoration. Earth-Sci. Rev..

[27] Lu X (2014). Effect of vegetation types on chemical and biological properties of soils of karst ecosystems. Eur. J. Soil Biol..

[28] Li D (2017). Dynamics of soil organic carbon and nitrogen following agricultural abandonment in a karst region. J. Geophys. Res.-Biogeosci..

[29] Li D (2018). Responses of soil nutrients and microbial communities to three restoration strategies in a karst area, southwest China. J. Environ. Manag..

[30] Hu N, Lan J (2020). Impact of vegetation restoration on soil organic carbon stocks and aggregates in a karst rocky desertification area in southwest China. J. Soils Sedim..

[31] Wang M (2018). Soil nutrients and stoichiometric ratios as affected by land use and lithology at county scale in a karst area, southwest China. Sci. Total Environ..

[32] Pincam T (2017). Hybrid Napier grass as a candidate species for bio-energy in plant-based water treatment systems: interactive effects of nitrogen and water depth. Aquat. Bot..

[33] Das A (2016). Impact of fodder grasses and organic amendments on productivity and soil and crop quality in a subtropical region of eastern Himalayas India. Agric. Ecosyst. Environ..

[34] Gartzia-Bengoetxea N (2009). Soil organic matter in soil physical fractions in adjacent semi-natural and cultivated stands in temperate Atlantic forests. Soil Biol. Biochem..

[35] Carter MR, Gregorich EG (2006). Soil Sampling and Methods of Analysis.

[36] Wang S, Li T, Zheng Z (2018). Tea plantation age effects on soil aggregate-associated carbon and nitrogen in the hilly region of western Sichuan China. Soil Tillage Res..

[37] Cheng Q (2020). Sediment sources, soil loss rates and sediment yields in a Karst plateau catchment in southwest China. Agric. Ecosyst. Environ..

[38] Liu M, Han G, Zhang Q (2020). Effects of agricultural abandonment on soil aggregation, soil organic carbon storage and stabilization: results from observation in a small karst catchment, southwest China. Agric. Ecosyst. Environ..

[39] Wang Z (2015). Soil organic carbon sequestration potential of artificial and natural vegetation in the hilly regions of Loess Plateau. Ecol. Eng..

[40] Gregory AS (2022). High-yielding forage grass cultivars increase root biomass and soil organic carbon stocks compared with mixed-species permanent pasture in temperate soil. Eur. J. Soil Sci..

[41] Sokolowski AC (2020). Tillage and no-tillage effects on physical and chemical properties of an Argiaquoll soil under long-term crop rotation in Buenos Aires, Argentina. Int. Soil Water Conserv. Res..

[42] Weidhuner A (2021). Tillage impacts on soil aggregation and aggregate-associated carbon and nitrogen after 49 years. Soil Tillage Res..

[43] Zhang X (2017). Effects of tillage and residue managements on organic C accumulation and soil aggregation in a sandy loam soil of the North China Plain. Catena.

[44] Zhao J (2017). Aggregate stability and size distribution of red soils under different land uses integrally regulated by soil organic matter, and iron and aluminum oxides. Soil Tillage Res..

[45] Xiao S (2017). Soil aggregate mediates the impacts of land uses on organic carbon, total nitrogen, and microbial activity in a Karst ecosystem. Sci. Rep..

[46] Kohler J (2017). Unraveling the role of hyphal networks from arbuscular mycorrhizal fungi in aggregate stabilization of semiarid soils with different textures and carbonate contents. Plant Soil.

[47] Daynes CN (2013). Development and stabilisation of soil structure via interactions between organic matter, arbuscular mycorrhizal fungi and plant roots. Soil Biol. Biochem..

[48] Zheng JY (2021). Soil aggregates are key factors that regulate erosion-related carbon loss in citrus orchards of southern China: bare land versus grass-covered land. Agric. Ecosyst. Environ..

[49] Zeng Q (2018). Soil aggregate stability under different rain conditions for three vegetation types on the Loess Plateau (China). Catena.

[50] LeBissonnais Y (1996). Aggregate stability and assessment of soil crustability and erodibility. 1. Theory and methodology. Eur. J. Soil Sci..

[51] Ni J (2015). Vegetation in karst terrain of southwestern China allocates more biomass to roots. Solid Earth..

[52] Hu D (2019). Seasonal changes and vertical distribution of fine root biomass during vegetation restoration in a karst area, southwest China. Front. Plant Sci..

[53] Gunina A, Kuzyakov Y (2014). Pathways of litter C by formation of aggregates and SOM density fractions: implications from 13C natural abundance. Soil Biol. Biochem..

[54] Urbanek E, Smucker AJM, Horn R (2011). Total and fresh organic carbon distribution in aggregate size classes and single aggregate regions using natural 13C/12C tracer. Geoderma.

[55] Kushwaha CP, Tripathi SK, Singh KP (2001). Soil organic matter and water-stable aggregates under different tillage and residue conditions in a tropical dryland agroecosystem. Appl. Soil Ecol..

[56] Tisdall JM, Oades JM (1982). Organic-matter and water-stable aggregates in soils. J. Soil Sci..

