# Supplementary material for: Dynamics of aggregate-associated organic carbon after long-term cropland conversion in a karst region, southwest China
Source: Sci Rep. 2023 Jan 31;13:1773. doi: 10.1038/s41598-022-27244-1 (PMC9889731; doi:10.1038/s41598-022-27244-1)
Supplement: Supplementary file 1 — Supplementary Tables. [file 41598_2022_27244_MOESM1_ESM.docx]

Table S1 Main effects of cropland use type and soil depth on soil OC content and stock of bulk soil and associated aggregate fractions.

|  |  | MS | SG | MB | FG |
| --- | --- | --- | --- | --- | --- |
| OC content  (g kg^-1^) | Bulk soil | 9.16 (0.79)b | 9.06 (0.90)b | 8.99 (0.77)b | 13.12 (1.25)a |
|  | >2 mm | 9.23 (0.88)b | 8.89 (0.88)b | 8.21 (0.91)b | 13.86 (1.32)a |
|  | 2–1 mm | 9.93 (0.91)b | 9.21 (0.83)b | 8.60 (0.85)b | 14.36 (1.51)a |
|  | 1–0.5 mm | 10.44 (0.94)b | 9.13 (0.90)b | 9.07 (0.92)b | 15.56 (1.92)a |
|  | 0.5–0.25 mm | 11.46 (1.17)b | 9.74 (0.94)b | 9.62 (1.03)b | 16.41 (2.04)a |
|  | <0.25 mm | 11.95 (1.42)b | 10.03 (0.93)b | 9.76 (1.09)b | 16.21 (1.92)a |
| OC stock  (Mg hm^-2^) | Bulk soil | 15.77 (1.85)b | 15.06 (1.09)b | 15.90 (1.85)b | 21.94 (2.66)a |
|  | >2 mm | 11.31 (1.18)b | 10.58 (0.75)b | 9.72 (1.10)b | 17.82 (1.44)a |
|  | 2–1 mm | 1.56 (0.19)b | 1.49 (0.18)b | 1.21 (0.17)b | 2.22 (0.23)a |
|  | 1–0.5 mm | 0.48 (0.09) | 0.51 (0.10) | 0.45 (0.08) | 0.53 (0.07) |
|  | 0.5–0.25 mm | 0.45 (0.10) | 0.52 (0.11) | 0.61 (0.14) | 0.43 (0.06) |
|  | <0.25 mm | 0.75 (0.17)b | 1.11 (0.15)ab | 1.46 (0.30)a | 0.79 (0.11)b |
|  |  |  |  |  |  |
|  |  | 0–10 cm | 10–20 cm | 20–30 cm |  |
| OC content  (g kg^-1^) | Bulk soil | 14.19 (0.87)a | 10.83 (0.68)b | 8.42 (0.80)c |  |
|  | >2 mm | 14.19 (0.94)a | 10.14 (0.79)b | 8.64 (0.86)bc |  |
|  | 2–1 mm | 14.86 (1.05)a | 10.84 (0.74)b | 8.82 (0.83)bc |  |
|  | 1–0.5 mm | 16.24 (1.36)a | 11.11 (0.79)b | 9.14 (0.92)bc |  |
|  | 0.5–0.25 mm | 17.64 (1.48)a | 11.83 (0.87)b | 9.53 (0.94)bc |  |
|  | <0.25 mm | 17.63 (1.54)a | 12.08 (0.94)b | 9.94 (0.96)bc |  |
| OC stock  (Mg hm^-2^) | Bulk soil | 17.72 (0.90)a | 14.52 (0.96)b | 11.35 (0.87)c |  |
|  | >2 mm | 13.20 (1.12)a | 10.29 (0.97)b | 9.67 (0.91)b |  |
|  | 2–1 mm | 2.02 (0.21)a | 1.57 (0.23)ab | 1.12 (0.15)b |  |
|  | 1–0.5 mm | 0.69 90.11)a | 0.48 (0.09)ab | 0.32 (0.03)b |  |
|  | 0.5–0.25 mm | 0.76 (0.15)a | 0.47 (0.09)ab | 0.32 (0.04)b |  |
|  | <0.25 mm | 1.52 (0.27)a | 0.97 (0.14)ab | 0.73 90.16)b |  |

Values presented as means with standard error. Different letters represented significant difference at *p* < 0.05 among four cropland types or three soil depth. MS: maize-soybean, SG: sugarcane, MB: mulberry, FG: forage grass.

Table S2 Changes in OC stocks (Mg/hm^2^) in each aggregate class at 0–30 cm depths of various cropland uses.

| Aggregate fractions | Variables | Changes in OC associated with aggregates at 0–10 cm depth | | | | Changes in OC associated with aggregates at 10–20 cm depth | | | | Changes in OC associated with aggregates at 20–30 cm depth | | | | Changes in OC associated with aggregates at 0–30 cm depth | | | |
| --- | --- | --- | --- | --- | --- | --- | --- | --- | --- | --- | --- | --- | --- | --- | --- | --- | --- |
|  |  | MS | SG | MB | FG | MS | SG | MB | FG | MS | SG | MB | FG | MS | SG | MB | FG |
| Macroaggregates  (>2 mm) | Mean value | 12.07 | 10.51 | 11.11 | 19.12 | 9.49 | 10.35 | 6.59 | 15.00 | 8.83 | 9.40 | 7.62 | 13.93 | 10.13 | 10.09 | 8.61 | 16.33 |
|  | Increment (Mg hm^-^2) | - | -1.56 | -0.96 | 7.05 | - | 0.86 | -2.90 | 5.51 | - | 0.57 | -1.21 | 5.10 | - | -0.04 | -1.52 | 6.20 |
|  | Incrementary ratio (%) | - | -12.92 | -7.95 | 58.41 | - | 9.12 | -30.58 | 58.10 | - | 6.46 | -12.88 | 66.99 | - | -0.40 | -15.03 | 61.21 |
| 2–1 mm | Mean value | 2.12 | 1.90 | 1.72 | 2.35 | 1.33 | 1.50 | 1.23 | 2.33 | 1.30 | 0.86 | 0.77 | 1.71 | 1.58 | 1.42 | 1.24 | 2.15 |
|  | Increment (Mg hm^-^2) | - | -0.23 | -0.40 | 0.22 | - | 0.17 | -0.10 | 1.00 | - | -0.44 | -0.53 | 0.41 | - | -0.17 | -0.34 | 0.57 |
|  | Incrementary ratio (%) | - | -10.72 | -18.85 | 10.48 | - | 12.99 | -7.34 | 75.52 | - | -34.04 | -61.52 | 53.51 | - | -10.47 | -21.51 | 35.85 |
| 1–0.5 mm | Mean value | 0.67 | 0.79 | 0.72 | 0.59 | 0.36 | 0.53 | 0.49 | 0.56 | 0.38 | 0.27 | 0.29 | 0.36 | 0.47 | 0.53 | 0.50 | 0.51 |
|  | Increment (Mg hm^-^2) |  | 0.12 | 0.05 | -0.09 | - | 0.17 | 0.12 | 0.19 | - | -0.11 | -0.10 | -0.02 | - | 0.06 | 0.03 | 0.04 |
|  | Incrementary ratio (%) |  | 17.47 | 7.06 | -12.64 | - | 46.21 | 34.25 | 53.56 | - | -29.61 | -35.51 | -5.85 | - | 12.19 | 5.62 | 8.34 |
| 0.5–0.25 mm | Mean value | 0.63 | 0.83 | 1.07 | 0.52 | 0.33 | 0.51 | 0.66 | 0.41 | 0.35 | 0.28 | 0.35 | 0.28 | 0.44 | 0.54 | 0.69 | 0.41 |
|  | Increment (Mg hm^-^2) | - | 0.21 | 0.44 | -0.11 | - | 0.19 | 0.33 | 0.09 | - | -0.08 | -0.01 | -0.07 | - | 0.11 | 0.26 | -0.02 |
|  | Incrementary ratio (%) | - | 32.54 | 41.75 | -10.38 | - | 57.69 | 102.05 | 26.15 | - | -21.99 | -1.82 | -20.86 | - | 24.14 | 59.67 | -4.83 |
| Microaggregates  <0.25 mm | Mean value | 1.06 | 1.61 | 2.34 | 1.05 | 0.64 | 1.06 | 1.48 | 0.77 | 0.60 | 0.73 | 1.02 | 0.51 | 0.76 | 1.13 | 1.63 | 0.81 |
|  | Increment (Mg hm^-^2) | - | 0.55 | 1.28 | -0.01 | - | 0.43 | 0.84 | 0.14 | - | 0.13 | 0.43 | -0.09 | - | 0.37 | 0.86 | 0.04 |
|  | Incrementary ratio (%) | - | 52.12 | 120.99 | -0.71 | - | 66.93 | 132.55 | 21.78 | - | 22.27 | 58.42 | -8.33 | - | 48.47 | 112.94 | 5.59 |

Table S3 The effects of cropland conversion on root biomass and root C content.

|  | MS | SG | MB | FG |
| --- | --- | --- | --- | --- |
| Root biomass  （kg hm^-2^） | 110.06 (17.24)c | 254.89 (55.89)bc | 568.95 (182.34)b | 660.83 (192.82)a |
| Root C  (kg hm^-2^) | 49.04 (7.74)c | 111.60 (25.36)bc | 258.71 (86.49)b | 292.53 (88.39)a |

Values presented as means with standard error. Different letters represented significant difference at *p* < 0.05 among cropland use types. MS: maize-soybean, SG: sugarcane, MB: mulberry, FG: forage grass.
